# The Genetic Polymorphisms of HLA Are Strongly Correlated with the Disease Severity after Hantaan Virus Infection in the Chinese Han Population

**DOI:** 10.1155/2012/308237

**Published:** 2012-10-08

**Authors:** Ying Ma, Bin Yuan, Jing Yi, Ran Zhuang, Jiuping Wang, Yun Zhang, Zhuwei Xu, Yusi Zhang, Bei Liu, Chao Wei, Chunmei Zhang, Angang Yang, Boquan Jin

**Affiliations:** ^1^Department of Immunology, The Fourth Military Medical University, 169 Changle West Road, Xi'an 710032, China; ^2^Department of Blood Transfusion, Xijing Hospital, The Fourth Military Medical University, Xi'an 710032, China; ^3^Department of Infectious Disease, Tangdu Hospital, The Fourth Military Medical University, Xi'an 710032, China; ^4^Department of Dermatology, Xijing Hospital, The Fourth Military Medical University, Xi'an 710032, China

## Abstract

The polymorphism of human leukocyte antigen (HLA), which is a genetic factor that influences the progression of hemorrhagic fever with renal syndrome (HFRS) after Hantaan virus (HTNV) infection, was incompletely understood. In this case-control study, 76 HFRS patients and 370 healthy controls of the Chinese Han population were typed for the HLA-A, -B, and -DRB1 loci. The general variation at the HLA-DRB1 locus was associated with the onset of HFRS (*P* < 0.05). The increasing frequencies of HLA-DRB1∗09 and HLA-B∗46-DRB1∗09 in HFRS patients were observed as reproducing a previous study. Moreover, the HLA-B∗51-DRB1∗09 was susceptible to HFRS (*P* = 0.037; OR = 3.62; 95% CI: 1.00–13.18). The increasing frequencies of HLA-B∗46, HLA-B∗46-DRB1∗09, and HLA-B∗51-DRB1∗09 were observed almost in severe/critical HFRS patients. The mean level of maximum serum creatinine was higher in HLA-B∗46-DRB1∗09 (*P* = 0.011), HLA-B∗51-DRB1∗09 (*P* = 0.041), or HLA-B∗46 (*P* = 0.011) positive patients than that in the negative patients. These findings suggest that the allele HLA-B∗46 and haplotypes HLA-B∗46-DRB1∗09 and HLA-B∗51-DRB1∗09 in patients could contribute to a more severe degree of HFRS and more serious kidney injury, which improve our understanding of the HLA polymorphism for a different outcome of HTNV infection.

## 1. Introduction

Hantaan virus (HTNV), the prototype member of the Hantavirus genus, causes hemorrhagic fever with renal syndrome (HFRS) in humans. Hantaviruses are enveloped negative-sense RNA viruses that belong to the family Bunyaviridae. They are rodent-borne viruses and are transmitted to humans primarily from aerosols of rodent excreta [1, 2]. People who get infected with HTNV are clinically characterized by sudden fever, hemorrhage, thrombocytopenia, and acute renal failure, leading from an asymptomatic to a severe, life-threatening illness. The pathogenic HTNV infection emerged as an increasing threat to human health with 60,000–100,000 cases worldwide reported annually and a case fatality rate of 6.04% [3, 4]. China is a severe epidemic area in which the numbers of HFRS cases and deaths remain the highest in the world [4]. However, the mechanisms underlying the different severities of HFRS are not yet understood.

It was proposed that the human leukocyte antigen (HLA) molecules are the host factors which might influence the status of disease. The HLA genetic system is encoded by the major histocompatibility complex (MHC) and is located at several loci on the short arm of the chromosome 6 in humans [5, 6]. HLA are highly polymorphic transmembrane glycoproteins that are expressed on the surface of lymphocytes [7]. Both HLA class I (-A, -B, and -C) and II (-DR, -DQ, and -DP) molecules are important determinants to be considered while presenting the antigenic epitopes to the host T lymphocytes in order to activate immune response [8]. However, the T-cell receptors are restricted by HLA molecules when they recognize the epitope peptides, which induce various interactions between epitopes and the host immune system [9]. Therefore, a host HLA genetic background is considered a risk factor that contributes to disease development.

The expression of HLA class I and II molecules in infected cells is observed either increase or decrease after virus infection [10–14], which suggests that the HLA-related host immunity could be responsible for not only the disease progression but also the controlling infection itself. In fact, several studies have focused on the correlation between the HLA allele polymorphism and the severity of hantaviruses infection. In Europe, the clinical severity of Puumala virus (PUUV-) induced nephropathia epidemica in humans depends on the HLA genetic background of the patients, with HLA-B*27 being associated to a benign clinical course and extended haplotypes HLA-B*8-DR*3 and HLA-DRB1*0301 being associated with severe patients [15, 16]. In the study of Slovenian population, Dobrava virus-infected patients have a significantly higher frequency of HLA-B*35 than PUUV-infected patients, whereas HLA-DRB1*13 was more frequent in PUUV-infected patients, especially in the severe form of the disease, and HLA-B*07 could play a protective role in PUUV-caused HFRS [17]. With regard to the hantavirus pulmonary syndrome (HPS) in North and South America, which is caused mainly by Sin nombre virus (SNV) and Andes virus, a correlation was observed between the presence of HLA-B*3501 and increased severity of disease symptoms after SNV infection [18], whereas the HLA-DRB1*15 was significantly more common in patients with mild disease, and HLA-B*08 was more common in patients with severe HPS after Andes virus infection in Chile [19]. As for other virus infectious diseases, the frequency of A*24 with histidine at codon 70 (A*2402/03/10) was significantly higher, and the HLA-DRB1*0901 frequency was significantly decreased in the dengue virus-infected Vietnamese population [20]. In another large case-control study of Vietnamese children, a variation at the HLA-A locus was significantly associated with susceptibility to dengue hemorrhagic fever; specifically, children with HLA-A*33 were less likely to develop dengue hemorrhagic fever, and children with the HLA-A*24 allele were at an increased risk of developing dengue hemorrhagic fever [21]. Our previous research has found out that there was a significantly higher occurrence of HLA-DRB1*09 and HLA-B*46-DRB1*09 in HFRS patients compared with the control group, indicating that HTNV-induced HFRS is associated with a genetic predisposition in the Chinese Han population [22]. However, there has been no evidence that illustrates the role of HLA allele polymorphisms in the degree of disease severity after HTNV infection in the Chinese Han population.

In this present study, we performed a case-control study that investigates the variation and specific polymorphism in HLA-A, -B, and -DRB1 of HTNV-infected patients, and explores the relationship between distinct alleles or haplotypes and the disease progression of HFRS in the Chinese Han population. The HLA gene polymorphisms especially in the DRB1 locus were found to be associated with HFRS. A novel haplotype was found to be susceptible to HFRS, and certain alleles and haplotypes would correlate to a great extent with the different severities of the disease.

## 2. Materials and Methods

### 2.1. Study Population

A total of 76 HFRS patients were enrolled in this study. The population consisted of an unrelated Han ethnicity that was recruited from the Department of Infectious Diseases at Tangdu Hospital of the Fourth Military Medical University at Xi'an, Northwest of China, during the period from September 2009 to January 2011. HTNV infection of the patients was diagnosed by clinical symptoms and history at the time of admission. The subjects were clinically diagnosed as mild or moderate patients when the mild renal failure without an obvious oliguric stage or obvious symptoms of uremia, effusion (bulbar conjunctiva), hemorrhage (skin and mucous membrane), and renal failure with a typical oliguric stage were observed [23]. The criteria of severe or critical infection were as follows: severe patients with severe uremia, effusion (bulbar conjunctiva and either peritoneum or pleura), hemorrhage (skin and mucous membrane), and renal failure with oliguria (urine output, 50–500 mL/day) for ≤5 days or anuria (urine output, <50 mL/day) for ≤2 days, and critical ones with ≥1 of the following symptoms during severe disease: refractory shock, visceral hemorrhage, heart failure, pulmonary edema, brain edema, severe secondary infection, and severe renal failure with either oliguria (urine output, 50–500 mL/day) for >5 days, anuria (urine output, <50 mL/day) for >2 days, or a blood urea nitrogen level of >42.84 mmol/L [23]. The genetic information on 370 demographic-matched healthy unrelated donors who had no history or symptoms of HTNV infection was collected randomly from the Center of Blood Transfusion at the Xijing Hospital of the Fourth Military Medical University (Xi'an, China) as a background population control group for the genetic study. No significant gender or age difference between the HFRS patients and healthy control individuals was observed.

Written informed consent was obtained from the patients and donors. The Institutional Review Board at the Tangdu Hospital and the Fourth Military Medical University approved the study.

### 2.2. Sample Collection and Serological Diagnosis

The blood samples were collected from each patient at the time of enrolment to the study during hospitalization. The whole blood was used for the DNA extraction. Clinical information, including age, gender, medical history, platelet counts, serum creatinine values, and so on, were recorded for each patient.

All samples were further confirmed for HTNV infection via serological testing of specific immunoglobulin M (IgM) and IgG with capture enzyme-linked immunosorbent assay in acute phase of the plasma.

### 2.3. Human DNA Extraction

The genomic DNA was extracted from the whole blood of the patients by using DAN^zol^ (Invitrogen, Carlsbad, CA) following the manufacturer's instructions [24]. Briefly, 1 mL DAN^zol^ was added to 100 *μ*L of whole blood and pipetted up and down gently to lyse the cells. Then, the lysate was centrifuged for 10 min at 10,000 ×g at 4°C, and the supernatant was transferred to a fresh tube. After that, 0.5 mL of absolute ethanol was added at room temperature for 3 min for the precipitation of DNA from the lysate. After a wash with 1 mL of 75% ethanol, DNA was solubilized in sterile water at a concentration of 50–150 ng/*μ*L for use. The values of A_260_ and A_280_ of the DNA solution were measured, and the A_260_/A_280_ ratio of the isolated DNA was within the 1.6–1.9 range.

### 2.4. HLA Typing

With regard to the samples in our case-control study, we determined HLA genotypes of all the study subjects using polymerase chain reaction (PCR-) based micro SSP DNA typing trays (One Lambda Inc., Canoga Park, CA) for HLA class I and II alleles according to standard procedures [25]. Briefly, preoptimized primers were dried and presented in different wells of a 96-well tube tray. DNA samples were added immediately after isolation to each well for PCR in the presence of recombinant Taq polymerase (One Lambda Inc., Canoga Park, CA, 5 units/*μ*L) and specially formulated dNTP-buffer mix (Micro SSP D-mix). Each typing tray included a negative control reaction tube that detects the presence of the internal control PCR product. PCR amplification was carried out in a 96-well thermal cycler. The program involved heating to 96°C for 130 seconds and to 63°C for 60 seconds, followed by 9 cycles of 96°C for 10 seconds and of 63°C for 60 seconds. Then, it involved 20 cycles of 96°C for 10 seconds, 59°C for 50 seconds, and 72°C for 30 seconds, and finally stopped at 4°C. After the PCR program, 2.5% agarose gel in the Micro SSP Gel System was used to electrophorese the samples. The presence or absence of specific PCR products was documented using the UV transilluminator. Interpretations of the typing were done with the lot-specific interpretation and specificity tables. HLA-A, HLA-B, and HLA-DRB1 genotype distributions were checked with the Hardy-Weinberg equilibrium.

### 2.5. Statistical Analysis

To analyze the association between allele prevalence and the severity of HTNV infection, the phenotype frequencies of HLA class I (HLA-A and -B) and II (HLA-DRB1) alleles were calculated by the direct counting method. Mean and 95% confidence intervals (CI) are utilized for skew-distributed continuous variables, and percentages are used for categorical variables to describe the data. The Mann-Whitney *U* test or the Student's *t*-test was used to compare the clinical parameters between groups according to the result of the Kolmogorov-Smirnov's normality test. The frequencies of individuals carrying particular alleles or haplotypes were compared between the two HFRS-infected patients group (mild/moderate and severe/critical), or between the HFRS group and the population background group, using the Chi-square test. Logistic regression analysis was carried out to calculate the odds ratios (ORs) and their 95% CI to identify the HLA loci that contribute to the onset of HFRS and to estimate the associations between HLA alleles or haplotypes and disease severity. Groups with higher OR values are suggestive of an increased risk of infection. Fisher's exact test is a statistical significance test that is used in the analysis of categorical data where sample sizes are small. *P* values were further subjected to correction according to the Bonferroni's inequality method (corrected* P*, *Pc*) by multiplying the *P* values with the number of alleles tested for each locus. *Pc* values less than 0.05 were considered statistically significant. The SPSS software package SPSS, version 14 (SPSS, Inc., Chicago), was used for these analyses.

## 3. Results

### 3.1. Characteristics of Study Population

The study was carried out at the Xi'an, Shaanxi Province of China. Seventy-six patients with confirmed HFRS infection were selected to investigate their HLA allele associations with the status of the disease. All the patients in this study were Chinese Han population and had the history of rodent contact. Forty-eight male and 28 female subjects, with a mean age of 42 years (range, 27 to 69), were diagnosed as mild HFRS in 13 patients, moderate HFRS in 22, severe HFRS in 27, and critical HFRS in 14 individuals. Considering the limited number of patients of each clinical type, patients with mild and moderate clinical types were combined and referred as a milder type group, and those with severe and critical types were ascribed to a more severe type group. The overall hospitalization period for patients with HTNV infection was 8–39 days. For the period of hospital admission, the blood urea nitrogen level, the serum creatinine concentration, and blood leukocyte counts were greatly elevated, whereas the serum albumin level decreased, and most of the patients underwent the thrombocytopenia. The patients with severe or critical type of HFRS all along had more severe clinical parameters than the mild or moderate ones. The clinical features of the study subjects are shown in Table 1.

### 3.2. Phenotype Frequencies of HLA-A, HLA-B, and HLA-DRB1 in HFRS Patients

We performed direct and comprehensive genotyping of 2 major HLA class I genes HLA-A and -B, and the major HLA class II gene, HLA-DRB1. Among the HFRS patients studied, 12 HLA-A alleles, 24 HLA-B alleles, and 12 HLA-DRB1 alleles were identified (Table  S1) (see Supplementary Material available online at doi:10.1155/2012/308237). The major alleles (phenotype frequencies having more than 5% in both the patient and healthy control groups) are shown in Table 2, which account for about 80–90% of the total phenotypes. We analyzed these major alleles for the evaluation of the risk of disease severity, because rare alleles would have little impact on population risk. The data of the healthy control group in our study were compatible with the earlier data on the same Chinese Han population living in the Guanzhong region of the Shannxi Province [26, 27].

### 3.3. The Major Association of HLA Loci Variation with HFRS

To reduce the loss of statistical power associated with HLA typing and correcting for numerous rare alleles at the HLA loci, the polymorphism was studied for HLA-A, B, and DRB1 mainly with regard to the higher-frequency alleles just identified in Table 2 and compared only these alleles for all HFRS patients and healthy control subjects. We initially assessed the overall effect of variation at the loci on the likelihood of developing HFRS by logistic regression analysis. The HLA-DRB1 loci consisted significantly of more polymorphisms in HFRS patients (*P* = 0.049), but the polymorphism in the HLA-A or -B genes showed no difference from the normal controls. These findings suggest that HLA-DRB1 was the major locus with polymorphisms that were probably related to the occurrence and disease progression of HFRS patients in the Chinese Han population.

### 3.4. The Association between the Variation in HLA Alleles and HFRS

A further analysis found that the polymorphism association of HLA-DRB1 loci is confined to two particular alleles (Table 3). People with the HLA-DRB1*09 allele were at an increased risk of developing HFRS (*P* = 0.004; *Pc* = 0.04; OR = 2.17; 95% CI: 1.27–3.69), whereas people with HLA-DRB1*12 (*P* = 0.031; *Pc* = 0.31; OR = 0.45; 95% CI: 0.22–0.94) were less likely to develop HFRS. With regard to the HLA class I loci, the frequencies of HLA-A alleles between HFRS patients and the control group were not significantly different, whereas certain alleles at HLA-B showed higher frequencies in HFRS patients. The frequency of HLA-B*46 in HFRS patients was positively associated with HFRS (*P* = 0.045; *Pc* = 0.540; OR = 1.84; 95% CI: 1.01–3.37) when compared with the control group (Table 3). However, only the difference still remained statistically significant for the allele DRB1*09 after Bonferroni correction, which was in accordance with our previous report [22].

### 3.5. The Association between the Variation in Haplotypes and HFRS

According to the study about the frequency of HLA haplotypes among the Guanzhong Han population, we observed six HLA A-B haplotypes, six HLA B-DRB1 haplotypes, and two HLA A-B-DRB1 haplotypes with frequencies higher than the others [27] and compared their frequencies between HFRS patients and normal controls. As shown in Table 4, neither the HLA A-B haplotypes nor the three loci haplotypes exhibited differences between patients and controls, whereas the two loci haplotypes HLA-B*46-DRB1*09 (*P* = 0.040; OR = 2.16; 95% CI: 1.02–4.55) and HLA-B*51-DRB1*09 (*P* = 0.037; OR = 3.62; 95% CI: 1.00–13.18) were more commonly shown in HFRS patients than controls.

### 3.6. The Association between Variation in HLA Alleles or Haplotypes and Disease Severity in HFRS

The HLA alleles or haplotypes just defined have only been found to be associated with HFRS. We next analyzed the contribution of the specific HLA alleles or haplotypes to the disease severity of HFRS patients. There were two groups: mild/moderate and severe/critical for comparison. As the data shown in Table 5, the comparison of the frequency between mild/moderate group and controls showed no difference for either the particular alleles or the haplotypes, whereas the significant differences in the frequency were observed between the severe/critical group and controls. Specifically, the frequencies of HLA-B*46 (*P* < 0.001; *Pc *< 0.001; OR = 3.44; 95% CI: 1.70–6.93), HLA-DRB1*09 (*P* = 0.006; *Pc* = 0.06; OR = 2.52; 95% CI: 1.29–4.93), HLA-B*46-DRB1*09 (*P* = 0.002; OR = 3.41; 95% CI: 1.48–7.86), and HLA-B*51-DRB1*09 (*P* = 0.016; OR = 4.92; 95% CI: 1.18–20.48) were much higher in severe/critical HFRS patients than in controls, and the frequency of HLA-DRB1*12 (*P* = 0.017; *Pc* = 0.17; OR = 0.26; 95% CI: 0.08–0.85) was much lower in the severe/critical group compared with controls, even though the *Pc* value of HLA-DRB1*09 and HLA-DRB1*12 just exceeded the significant level after Bonferroni's correction. Then, the comparison between mild/moderate and severe/critical groups showed that the frequency of HLA-B*46 was significantly lower in the milder group than in the more severe one. (*P* = 0.004; *Pc *= 0.018; OR = 0.16; 95% CI: 0.04–0.62), suggesting that the patients with HLA-B*46 were almost with a more severe form of HFRS. Next, the clinical parameters of the HFRS patients were analyzed and compared between the HLA allele and the haplotype-positive or -negative groups. As the results shown in Table 6, the mean level of maximum serum creatinine was significantly higher in patients with HLA-B*46-DRB1*09 (*P* = 0.011), or with HLA-B*51-DRB1*09 (*P* = 0.041), or with HLA-B*46 (*P* = 0.011) than in the allele or haplotype-negative group of patients. These findings pointed out that the more severe HFRS were strongly associated with haplotype HLA-B*46-DRB1*09 or HLA-B*51-DRB1*09 and the allele HLA-B*46.

## 4. Discussion

The host genetic factors have been considered to be associated with hundreds of human diseases. The HLA allele profile is one of the most important factors that could play a role in susceptibility or protection from diseases. It has been reported that the molecular mechanisms underlying many infectious diseases are associated with particular HLA molecules [19, 28–31] and the HLA allele-restricted cellular immune responses [32, 33]. For most of these diseases, it has often been difficult to unequivocally ascertain the primary disease-risk HLA genes, because the diseases are usually the result of the combination of different HLA molecules expressed at various loci (class-I and/or class-II) rather than the result of one HLA variant only [34]. Thus, although the distinct HLA allele associated with the HTNV-susceptible population was previously reported [22], the correlation between the HLA alleles or haplotypes and the disease severity still remains unclear. This study would be an effort to determine the certain alleles or haplotypes that could serve as a contributing factor in the progression of the severity of HFRS in the Chinese Han population.

The general analysis of the HLA-A, B, and DRB1 showed that the polymorphism of the HLA-DRB1 loci was the greatest during the HFRS. Among them, the frequencies of allele HLA-DRB1*09, and related haplotypes HLA-B*46-DRB1*09 and HLA-B*51-DRB1*09 were increased only in severe/critical HFRS cases. HLA-DR is the major gene that encodes the HLA class II products, which are considered as playing a crucial role in the presentation of processed antigen to specific CD4^+^ T cells. Our findings suggest that there might be various CD4^+^ T-cell responses induced by the HLA-DRB1 polymorphism which would contribute to the different severities of HFRS. The HLA-DRB1*09 restricted CD4^+^ T-cell response targeting one or several epitopes of HTNV would secret proinflammatory cytokines which may induce the immune injury. However, the study in the Syrian hamster model of HPS showed that the T-cell response was not required for pathogenesis of the disease [35]. Thus, further studies are still needed to clarify the possible mechanism between HLA polymorphism and the T-cell response-related severity of the disease.

The results of HLA association from the present study reproduced our previous study [22] in which HLA-DRB1*09 and HLA-B*46-DRB1*09 were found to be associated with HFRS. Considering such reproducibility in different study periods, within the same ethnic group and in the same region, give us the confidence to analyze the susceptible role of the HLA-DRB1*09 and HLA-B*46-DRB1*09 in HFRS. A further analysis showed that besides the allele HLA-DRB1*09 and haplotype HLA-B*46-DRB1*09, there are still other HLA alleles or haplotypes that are associated with the susceptibility of HFRS. The alleles HLA-B*46, HLA-B*51 and haplotype HLA-B*51-DRB1*09 increased strongly in frequency with the likelihood to contribute to the occurrence of HFRS when compared with the control group, whereas the frequency of HLA-DRB1*12 was lower in HFRS than the control, suggesting the role played by resistance from the onset of HFRS. The protective effect of this allele against HFRS could be considered a new finding in HTNV infection, although the *P* value exceeded 0.05 after Bonferroni's correction. The reason that these new alleles and haplotype were not identified in our previous study could primarily be due to the insufficient sample size. Since the HFRS patients infected by HTNV appear only 3 to 4 months during autumn and winter [36], the samples could be obtained from the limited number of HFRS patients. If we could increase the number of patients by studying their HLA polymorphism, then there would probably be more significant results after Bonferroni's correction for HLA-B*46, HLA-B*51, or HLA-DRB1*12. However, this study was still considered a good complement to the comprehensive investigation of the HLA polymorphism during HFRS.

Important clinical findings in this study showed that the particular HLA alleles or haplotypes we defined could contribute to the disease severity after HTNV infection. Since the kidney was the primary organ that was seriously injured during HFRS, the elevated serum creatinine could directly reflect the kidney injury and the degree of disease severity. The contribution was confirmed by a correlation between the HLA-B*46, HLA-B*46-DRB1*09, or HLA-B*51-DRB1*09 positive patients and the significantly higher level of serum creatinine compared with these allele or haplotype-negative patients. Moreover, the significant difference in the frequency was usually observed between the severe/critical patients group, but not the mild/moderate group, and controls for the allele HLA-B*46 and haplotype HLA-B*46-DRB1*09 or HLA-B*51-DRB1*09. These results, therefore, agree with the conclusion that the patients with a particular HLA allele or haplotype (HLA-B*46, HLA-B*46-DRB1*09, or HLA-B*51-DRB1*09) would be correlated with a more severe form of HFRS.

## 5. Conclusion

In conclusion, our study confirmed the previous reported HLA association between allele HLA-DRB1*09, haplotype HLA-B*46-DRB1*09, and the onset of HFRS. Moreover, we showed that the people with the haplotype HLA-B*51-DRB1*09 were also susceptible to HFRS, and the allele HLA-B*46 and haplotypes HLA-B*46-DRB1*09 and HLA-B*51-DRB1*09 in patients could contribute to a more severe degree of HFRS and more serious kidney injury. This study represents a further complementary study that improves our understanding of the risk of HLA for the severe outcome of HTNV infection. Identification of population-specific association alleles is critical for medicine. This information on HLA alleles would probably be useful in identifying appropriate epitopes for molecular vaccines and in determining the possible efficacy of these vaccines in the Chinese Han population.

## Supplementary Material

The phenotype frequencies of HLA-A, HLA-B, and HLA-DRB1 alleles in hemorrhagic fever with renal syndrome patients and healthy controls. There are 12 alleles at HLA-A locus, 24 alleles at HLA-B locus and 12 alleles at HLA-DRB1 locus, respectively.

## Figures and Tables

**Table 1:** Clinical characteristics of the study subjects.

		HFRS patients		Controls
	Total	Mild/Moderate	Severe/Critical
	*n* = 76	*n* = 35	*n* = 41	*n* = 370
Mean age (year)^a^	42 (40–44)	41 (38–44)	43 (40–46)	40 (39–41)
Male (%)^a^	63.2	57.1	68.3	59.5
Number of stages in HFRS^b^	3–5	3-4	4-5	—
Mean course (days)^c^	20 (18–21)	15 (14–16)**	24 (22–26)**	—
Mean S-BUN_max_ (mmol/L)	20.4 (17.9–22.9)	14.4 (11.6–17.3)**	25.6 (22.3–28.8)**	—
Mean S-Crea_max_ (*μ*mol/L)	444.0 (391.2–496.8)	367.2 (288.5–445.9)*	509.6 (442.0–577.2)*	—
Mean B-Leuk_max_ (*10^9^/L)	24.3 (20.3–28.3)	19.0 (15.5–22.6)*	28.8 (22.3–35.3)*	—
Mean B-Throm_min_ (*10^9^/L)	28.4 (23.1–33.6)	41.8 (33.3–50.3)**	16.9 (12.8–21.0)**	—
Mean S-ALB_min_ (g/L)	27.9 (26.6–29.2)	31.6 (30.1–33.2)**	24.7 (23.2–26.2)**	—

The continuous variables were presented as mean (95% confidence interval). The Mann-Whitney *U* test was used to compare the groups; the Chi-square test was used for the percentage of men. HFRS: hemorrhagic fever with renal syndrome. *n*: number of patients; S-BUN_max_: highest blood urea nitrogen; S-Crea_max_: highest serum creatinine concentration; B-Leuk_max_: highest blood leukocyte count; B-Throm_min_: lowest blood thrombocyte count; S-ALB_min_: lowest serum albumin concentration (all measured during hospital care).

^a^There were no differences pertaining to age and male percent between total HFRS patients and controls or between the milder group and the more severe group.

^b^The stages of HFRS include febrile, hypotensive, oliguric, diuretic, and convalescence.

^c^The days from fever onset to convalescence discharge from the hospital.

**P < 0.01 between the milder group and the more severe group.

*P < 0.05 between the milder group and the more severe group.

**Table 2:** The polymorphism and susceptibility of class I and II loci in the major histocompatibility complex to hemorrhagic fever with renal syndrome.

Class, locus	Alleles	Number of alleles studied	df	Residual *χ* ^2^	*P *
Class I					
HLA-A	*02,*11,*31,*30,*33,*32,*01,*24,*03	9	9	6.17	0.722
HLA-B	*13,*40,*15,*58,*51,*07,*46,*44,*35,*54,*48,*52	12	12	10.74	0.551
Class II					
HLA-DRB1	*07,*12,*04,*09,*11,*15,*14,*08,*03,*13	10	10	18.39	**0.049 **

The alleles were selected for frequency >5%. Logistic regression was used for analysis. Locus in bold indicates that its polymorphism is linked to susceptibility to hemorrhagic fever with renal syndrome. Number in bold indicates a significant *P* value.

**Table 3:** Comparison of the phenotype frequencies of major alleles of HLA-A, -B, and -DRB1 in hemorrhagic fever with renal syndrome population and control group.

Phenotype	HFRS	Controls	Fold of frequency	*P*	*Pc *	OR (95% CI)
	AF (%)	AF (%)				
HLA-B*46	24.32	14.86	1.64	0.045	0.540	1.84 (1.01–3.37)
HLA-DRB1*12	12.50	24.05	0.52	0.031	0.310	0.45 (0.22–0.94)
HLA-DRB1*09	38.89	22.70	1.71	0.004	0.040	2.17 (1.27–3.69)

The alleles were selected for a frequency >5% and the *P* value < 0.05. *P* value derived from Pearson Chi-square test; HFRS: hemorrhagic fever with renal syndrome; OR: odds ratio; CI: confidence interval; AF: allele frequency in percentage.

The frequencies of the following alleles are not significant different between HFRS and controls: HLA-A*02, *11, *31, *30, *33, *32, *01, *24, *03; HLA-B*13, *40, *15, *58, *51, *07, *44, *35, *54, *48, *52; HLA-DRB1*07, *04, *11, *15, *14, *08, *03, *13.

**Table 4:** Comparison of the haplotype frequencies in hemorrhagic fever with renal syndrome population and control group.

Haplotypes	HFRS patients	Controls	Fold of frequency	*P*	OR (95% CI)
	AF (%)	AF (%)			
HLA-B*46-DRB1*09	15.49	7.84	1.98	0.040	2.16 (1.02–4.55)
HLA-B*51-DRB1*09	5.63	1.62	3.47	0.037	3.62 (1.00–13.18)

The haplotypes were selected for a frequency >5% in either patients group or control group and the *P* value < 0.05. *P* value derived from Pearson Chi-square test; HFRS: hemorrhagic fever with renal syndrome; OR: odds ratio; CI: confidence interval; AF: allele frequency in percentage.

The frequencies of the following haplotypes are not significant different between HFRS and Controls: A*02-B*46, A*30-B*13, A*33-B*58, A*02-B*51, A*02-B*13, A*11-B*13, B*13-DRB1*07, B*13-DRB1*12, B*52-DRB1*15, B*13-DRB1*15, A*30-B*13-DRB1*07, A*02-B*46-DRB1*09.

**Table 5:** The association between alleles or haplotypes and severity types in hemorrhagic fever with renal syndrome.

Allele or haplotype	Fold of frequency	*P *	*Pc *	OR (95% CI)
HLA-B*46				
mild/moderate versus control	0.59	0.336		0.55 (0.16–1.88)
severe/critical versus control	2.52	**<0.001**	**<0.001**	**3.44 (1.70**–**6.93)**
mild/moderate versus severe/critical	0.24	**0.004**	**0.018**	**0.16 (0.04**–**0.62)**

HLA-DRB1*09				
mild/moderate versus control	1.51	0.136		1.78 (0.83–3.85)
severe/critical versus control	1.87	**0.006**	**0.06**	**2.52 (1.29**–**4.93)**
mild/moderate versus severe/critical	0.81	0.482		0.71 (0.27–1.85)

HLA-DRB1*12				
mild/moderate versus control	0.78	0.498		0.73 (0.29–1.83)
severe/critical versus control	0.31	**0.017**	**0.17**	**0.26 (0.08**–**0.85)**
mild/moderate versus severe/critical	2.50	0.151		2.85 (0.65–12.43)

HLA-B*46-DRB1*09				
mild/moderate versus control	0.82	0.781		0.81 (0.18–3.57)
severe/critical versus control	2.87	**0.002**	—	**3.41 (1.48**–**7.86)**
mild/moderate versus severe/critical	0.29	0.064		0.24 (0.05–1.19)

HLA-B*51-DRB1*09				
mild/moderate versus control	1.99	0.512		2.02 (0.24–17.35)
severe/critical versus control	4.63	**0.016**	—	**4.92 (1.18**–**20.48)**
mild/moderate versus severe/critical	0.43	0.439		0.41 (0.04–4.16)

The alleles and haplotypes were selected for their *P* value of frequency < 0.05 in Tables 3 and 4. *P* value derived from Pearson Chi-square test; OR: odds ratio; CI: confidence interval; numbers in bold indicate a significant *P* value.

**Table 6:** Comparison of the clinical laboratory findings in HLA allele or haplotype-positive and -negative hospitalized patients with hemorrhagic fever with renal syndrome.

Characteristics	HLA-B*46-DRB1*09 positive	HLA-B*46-DRB1*09 negative	*P *	HLA-B*51-DRB1*09 positive	HLA-B*51-DRB1*09 negative	*P *	HLA-B*46 positive	HLA-B*46 negative	*P *
Mean age (year)	44 (37–51)	42 (39–44)	0.428	46 (21–72)	42 (40–44)	0.334	43 (38–47)	42 (39–44)	0.360
Mean course (days)^a^	22 (17–27)	19 (18–21)	0.183	20 (12–29)	20 (18–21)	0.700	22 (18–25)	19 (17–21)	0.090
Mean s-BUN_max_ (mmol/L)	19.54 (11.06–28.02)	20.57 (17.94–23.20)	0.637	19.23 (4.41–42.88)	20.49 (17.97–23.01)	0.701	20.50 (14.32–26.69)	20.39 (17.65–23.14)	0.769
Mean s-Crea_max_ (*μ*mol/L)	596.01 (495.32–696.70)	418.30 (360.51–476.09)	**0.011 **	690.02 (383.47–1004.60)	430.13 (377.04–483.22)	**0.041 **	553.02 (469.03–637.00)	410.19 (347.53–472.86)	**0.011 **
Mean Leuk_max_ (*10^9^/L)	16.69 (13.13–20.25)	25.59 (21.05–30.13)	0.275	18.06 (11.10–25.01)	24.65 (20.48–28.82)	0.798	24.55 (14.25–34.86)	24.23 (19.93–28.52)	0.797
Mean PLT_min_ (*10^9^/L)^a^	48.36 (24.95–71.77)	34.97 (20.37–29.57)	0.073	43.00 (13.03–99.03)	27.54 (22.35–32.74)	0.334	39.33 (23.97–54.70)	24.95 (19.99–29.91)	0.087
Mean s-ALB_min_ (g/L)	30.36 (26.75–33.97)	27.46 (26.03–28.90)	0.125	30.03 (24.38–35.67)	27.77 (26.38–29.15)	0.451	28.98 (26.33–31.64)	27.54 (25.99–29.10)	0.766

The continuous variables were presented as mean (95% confidence interval). *P* value derived from the Mann-Whitney *U* test or the Student's *t*-test. S-BUN_max_: highest blood urea nitrogen; S-Crea_max_: highest serum creatinine concentration; B-Leuk_max_: highest blood leukocyte count; B-Throm_min_: lowest blood thrombocyte count; S-ALB_min_: lowest serum albumin concentration (all measured during hospital care). Numbers in bold indicate an almost significant *P* value.

^a^The days from fever onset to convalescence discharge from the hospital.
