# Supplementary material for: The Genetic Polymorphisms of HLA Are Strongly Correlated with the Disease Severity after Hantaan Virus Infection in the Chinese Han Population
Source: Clin Dev Immunol. 2012 Oct 8;2012:308237. doi: 10.1155/2012/308237 (PMC3472611; doi:10.1155/2012/308237)
Supplement: Supplementary file 1 — The phenotype frequencies of HLA-A, HLA-B, and HLA-DRB1 alleles in hemorrhagic fever with renal syndrome patients and healthy controls. There are 12 alleles at HLA-A locus, 24 alleles at HLA-B locus and 12 alleles at HLA-DRB1 locus, respectively. [file 308237.f1.pdf]

Table S1. Phenotype frequencies of HLA-A, HLA-B, and HLA-DRB1 alleles

Allele	HFRS patients			Controls
	Total	Mild/Moderate	Severe/Critical	
	AF(%)	AF(%)	AF(%)	AF(%)
HLA-A				
*01	9.21	11.43	7.32	8.65
*02	47.37	45.71	46.34	50.27
*03	9.21	5.71	12.20	7.84
*11	34.21	31.43	36.59	32.70
*23	1.32	2.86	-	0.81
*24	22.37	28.57	14.63	31.89
*26	3.95	2.86	4.88	4.59
*30	10.53	17.14	2.44	13.78
*31	6.58	5.71	7.32	7.30
*32	11.84	8.57	14.63	7.30
*33	15.79	14.29	17.07	12.97
*29	1.32	2.86	-	1.08
HLA-B				
*07	9.46	11.76	7.50	7.57
*13	17.57	26.47	7.50	21.89
*15	21.62	26.47	17.50	26.49

*18	2.70	5.88	-	1.35
*27	2.70	2.94	2.50	5.14
*35	10.81	17.65	5.00	11.62
*37	1.35	2.94	-	3.24
*38	4.05	5.88	2.50	5.14
*40	21.62	17.65	25.00	23.51
*41	2.70	2.94	2.50	-
*44	10.81	5.88	15.00	10.00
*46	24.32	8.82	37.50	15.95
*48	6.76	11.76	2.50	5.68
*50	1.35	-	2.50	3.24
*51	17.57	14.71	20.00	10.54
*52	8.11	2.94	12.50	8.65
*54	6.76	2.94	10.00	6.49
*55	2.70	-	5.00	5.41
*56	1.35	2.94	-	1.08
*57	4.05	-	7.50	4.32
*58	10.81	11.76	10.00	8.92
*81	1.35	2.94	-	-
*62	2.70	5.88	-	-
*67	1.35	-	2.50	2.16

HLA-DRB1

*01	4.17	3.13	5.00	6.76
*03	11.11	9.38	12.50	8.65
*04	20.83	9.38	30.00	24.05
*07	29.17	37.50	22.50	22.70
*08	13.89	18.75	10.00	8.92
*09	38.89	34.38	42.50	22.70
*11	11.11	15.63	7.50	15.68
*12	12.50	18.75	7.50	24.05
*13	5.56	9.38	2.50	9.19
*14	9.72	12.50	7.50	12.97
*15	25.00	12.50	35.00	30.00
*16	5.56	3.13	7.50	1.89

HLA, human leukocyte antigen; AF, allele frequency (as percentage); HFRS, hemorrhagic fever with renal syndrome.
